# Multi‐scale Optimization on Interfacial Evaporative Cooling for Photovoltaic Performance Enhancement

**DOI:** 10.1002/advs.75120

**Published:** 2026-04-07

**Authors:** Fuxiang Li, Haosheng Lin, Zengguang Sui, Yunren Sui, Zhixiong Ding, Wei Wu

**Affiliations:** ^1^ School of Energy and Environment City University of Hong Kong Hong Kong China; ^2^ City University of Hong Kong Shenzhen Research Institute Shenzhen China

**Keywords:** interfacial evaporation, multiphysics model, multiscale optimization, photovoltaic, thermal management

## Abstract

Over 70% of incident sunlight is wasted as heat in silicon photovoltaics (PV), raising operating temperatures and degrading performance. This study proposes efficient interfacial evaporation‐based cooling technology and systematically optimizes it towards array‐scale applications. Research confirms the evaporator's water transport capacity far exceeds the thermal load (∼1200 W/m^2^ max). A thin‐film evaporator achieves superior cooling, reducing PV temperature by nearly 18 °C. A developed multiphysics model shows excellent agreement with experiments, accurately predicting PV temperature, electrical characteristics, and evaporation rate. Simulations reveal that a moisture boundary layer (MBL) forms on the PV backside; minimizing its thickness is key to enhancing cooling. Array‐level analysis demonstrates that simply increasing installation height in existing PV plants improves rear ventilation sufficiently. This approach achieves up to 22.3 °C temperature reduction and an 8.9% relative power efficiency gain without inducing electrical mismatch. The work provides both a theoretical foundation and practical pathways for efficient thermal management in PV.

## Graphical abstract

This study presents a passive siphon‐driven interfacial evaporative cooling technology for photovoltaics. A multiscale multiphysics model is developed and validated experimentally. Results show that optimizing installation parameters reduces operating temperature by up to 22.3 °C and boosts power efficiency by 8.9%, offering a practical pathway for array‐scale thermal management.

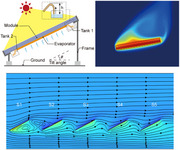

## 1 Introduction

Frequent climate extremes with surging population poses significant challenges to global sustainability [1]. Sustainable energy transition towards carbon neutrality is a key measure for sustainable development [2, 3]. Solar energy is critical in this transition from fossil fuels to renewable energy [4]. Photovoltaics (PV) modules witnessed a significant efficiency improvement and cost decline in recent decades [5]. The high cost‐effectiveness can compete with traditional power generation via fossil fuel [6]. Increasing the PV capacity has become the core strategy to achieve carbon‐neutrality in many countries [7]. The cumulative capacity of PV is projected to increase to 15 terawatt (TW) by the end of 2050 [8]. Beyond traditional large‐scale power plants, PV applications are expanding into buildings [9, 10, 11], transportation [12, 13, 14], agriculture [15], and space bases [16], creating synergistic opportunities across sectors.

Despite the promising prospect, the maximum theoretical efficiency of crystalline silicon (c‐Si) is 33.3% due to the Shockley–Quisser limit [17]. Currently, the champion c‐Si solar cell efficiency is 27%, which means nearly 70% of the incident solar energy is wasted as heat [18]. The resulting high operating temperature can adversely affect the PV electrical performance and module lifetime [19]. Taking the standard testing condition (STC) as the reference, the PV output has a temperature coefficient of 0.24–0.45%/°C [20]. Simultaneously, the high temperature also increases the failure rate of the PV [21]. These challenges underscore the importance of PV thermal management [22].

Existing PV thermal management strategies can be classified into active (i.e., need external energy input) and passive strategies (i.e., no external energy input) [23]. Active strategies typically use a fan or pump to drive fluid (air, water, nanofluid) for heat removal. Fast heat removal (reduce *T*_pv_ by 20 °C) can be achieved via enhanced fluid flow, while the cost is the huge power consumption of the fan/pump [24]. Additionally, the high initial cost due to the additional system configuration also unfavored the adoption of active technologies. Passive strategies, using functional materials to achieve spontaneous heat dissipation offers a promising pathway to this issue [25]. For example, radiative cooling (RC) can emit heat via radiation to the universe via the atmospheric window, thereby reducing the PV operating temperature [26]. However, theoretical temperature reduction is only 3‐5 °C due to an inherent constraint: the PV cell requires high solar absorption while enhanced thermal radiation can only provide limited cooling power [27, 28]. Phase change material (PCM) is another passive PV cooling strategy [29]. As it can maintain a stable temperature during the phase transition process, PCM can be packed under the PV module to absorb the waste heat. Unfortunately, the low thermal conductivity and cycle degradation are two unresolved bottlenecks [30]. Additionally, harnessing the naturally cool microclimates of surrounding environments is regarded as an efficient passive cooling strategy, as seen in floating PV and submerged PV systems [31, 32]. However, the reliance on water bodies in such approaches does not align with the actual installation conditions of most PV systems [33, 34]. Sorption‐based PV cooling technology, which captures atmospheric water vapor at night for daytime desorption cooling, has attracted significant attention in recent years due to its passive feature and high *T*_pv_ reduction (13–18 capillarity°C) [35, 36, 37, 38]. However, the technology is still in its early stages, and further efforts are needed to scale it up and improve its nocturnal adsorption recovery rate [39, 40].

Since water is the most common cooling medium, water‐based PV thermal management has consistently been a key research focus. On the front side of PV, methods such as spray cooling and water jetting are employed, which not only reduce the *T*_pv_ but also clean the glass surfaces [41, 42, 43]. To further minimize water consumption, recent studies have proposed evaporative cooling (EC) technologies utilizing porous material evaporators installed on the back of PV panels [44, 45]. In small PV panel, water can be supplied via capillary suction to sustain the evaporation [46, 47]. For large PV panels, a gravity‐driven water supply is a more suitable way [48]. Although the effectiveness of EC‐based systems has been adequately demonstrated in previous research, the underlying heat and mass transfer mechanism and its impact on electrical performance remain elusive. A comprehensive modeling framework that can estimate system performance and guide system design is still in high demand.

Additionally, most PV thermal management studies, including those on evaporative cooling (EC), are confined to the analysis of single panels or small‐scale prototypes. This constitutes a significant gap, as real‐world PV systems operate in array configurations where complex inter‐panel effects prevail. A typical array consists of several module strings, while each module string is made of several electrically interconnected multiple panels. As a result, PV arrays significantly influence the surrounding flow field and solar irradiation conditions [49]. This poses two key challenges for PV thermal management: first, whether the performance observed at the single‐panel level can be maintained within an array configuration; second, how to accurately analyze the performance of array‐scale PV systems with thermal management. Although some studies have addressed inter‐array spacing to mitigate mutual shading and enhance ventilation for heat convection [50, 51], none have taken into account the scenario with the presence of thermal management. To enable PV thermal management to serve practical PV systems, it is essential to account for cross‐scale effects and address thermal management issues in utility‐scale arrays.

Generally, there are three bottlenecks in existing studies that prevent the progress of the EC‐based PV thermal management. First, a profound mechanistic understanding of these systems remains limited, notably the multiphysics interactions among heat transfer, electrical performance, fluid flow, and moisture transport. Furthermore, the influencing factors on array‐level performance have not been systematically investigated, nor has a dedicated thermal management analysis methodology been established for such scales. Ultimately, from an engineering practice perspective, there is still a lack of guidance for array‐level thermal management.

To address these issues, this study proposes a multiscale approach combining multiphysics simulation and scaled experiments. Based on traditional EC‐based PV cooling, a novel PV thermal management technology called self‐adaptive water evaporative cooling (PV‐SWE) is proposed. This system uses the passive siphon effect to provide water for interfacial evaporation, which is zero energy and highly efficient. This research developed a multi‐scale, multiphysics coupling approach applicable at the evaporator, module, and array levels to identify the key factors governing performance at each scale. These models have been validated through meticulously scaled experiments. The results not only confirm the effectiveness of PV‐SWE as a novel cooling technology but also clarify the critical factors influencing its large‐scale application, thereby laying a solid foundation for full‐scale engineering implementation.

## 2 Results

### 2.1 Design and Fabrication of PV‐SWE

**FIGURE 1:**
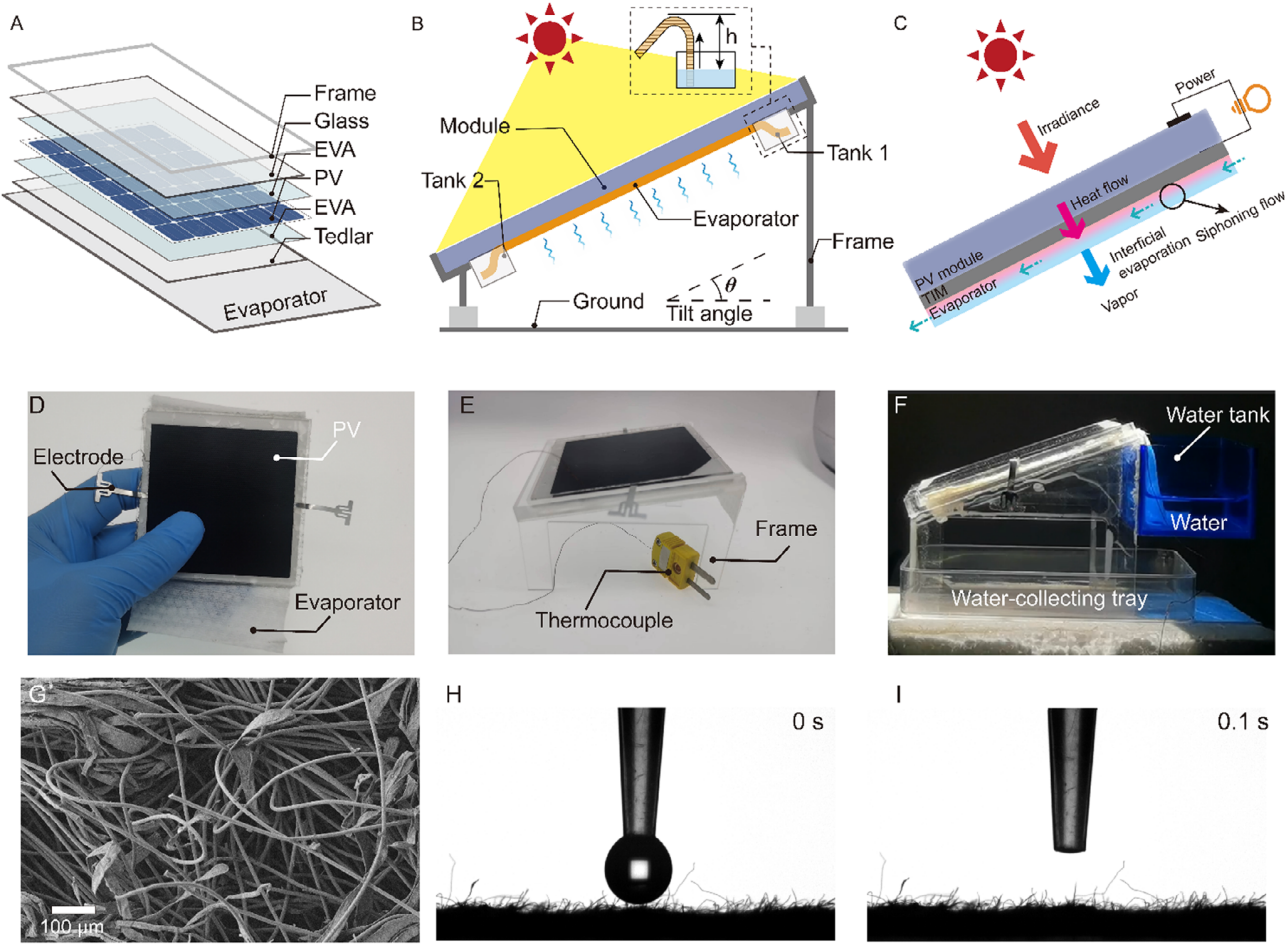
Schematic of the PV‐SWE system. (A) Tandem structure of the PV‐SWE. (B) Illustration of the working principle. (C) Energy conversion process in PV‐SWE, where the waste heat is rejected by the vapor flow. (D,E) Scaled PV‐SWE fabrication process. (G,H) The evaporator materials feature a porous structure and good hydrophilicity.

The PV‐SWE module retains the conventional, framed tandem structure of a standard PV module, which consists of glass, Ethylene Vinyl Acetate Copolymer (EVA), PV cell layer, EVA, and a Tedlar backsheet. An evaporator, made of a thin water‐absorbing layer (e.g., cotton fabric), is attached to the bottom of the module (Figure 1A). Similar to a standard PV module, it is mounted on a support frame at a specific tilt angle (*θ*) to maximize the incident sunlight for power generation (Figure 1B). Crucially, the evaporator extends slightly beyond the length of the module. Its upper end contacts a micro‐water tank, while its lower end connects to another collection tank. When the upper tank is filled with water and the upper tail of the evaporator contacts the water, driven by capillary action, water ascends against gravity to a height (*h*). Once the water surpasses the apex of the evaporator, gravity becomes the primary driving force, ultimately establishing a siphon flow. This process passively forms a thin water film on the back of the PV panel, which effectively removes waste heat through evaporation under sunlight, thereby helping maintain high PV efficiency (Figure 1C). The fabrication process of the PV‐SWE is similar to the standard PV lamination process, where the evaporator is bonded to the backsheet using an EVA or other adhesive interface layer with good thermal conductivity (Figure 1D). Following assembly, the prototype is secured to the frame. A thermocouple is integrated forPV temperature monitoring (Figure 1E). The upper water tank and the dual‐purpose water collection tray (functioning as the lower tank) are then affixed (Figure 1F). The evaporator component is characterized by its construction from hydrophilic cotton fabric (Figure 1G), a material selected for its porous architecture and high water absorbency (Figure 1H–I).

### 2.2 From Initiation to Sustained Operation

#### 2.2.1 Siphon Initiation and Maximum Sustainable Cooling Power

**FIGURE 2:**
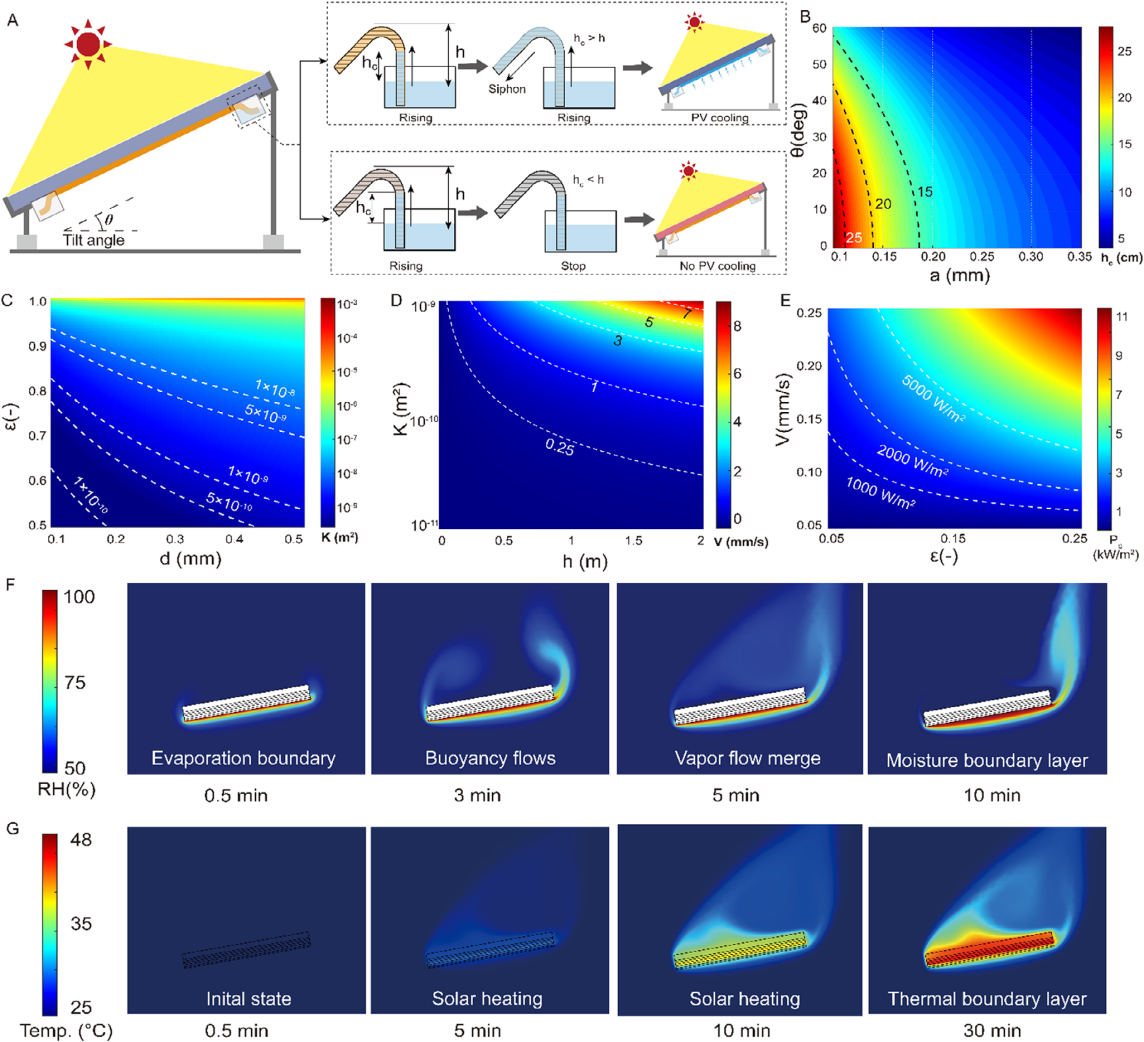
Theoretical analysis of the start‐up phase and steady‐state heat flux characteristics of the evaporator. (A) The initiation of the siphon effect. (B) Final height of the capillary water column for different evaporators. (C) Hydraulic conduction coefficient as determined by the Kozeny–Carman equation. (D) Siphon velocity under different height differences and hydraulic conduction coefficients. (E) Theoretical limit of evaporative cooling flux for a 3‐mm evaporator at different porosities and siphon velocities. (F) The development of the moisture boundary layer under PV‐SWE. (G) The development of the temperature field.

As indicated in Figure 2A, the stable operation of the PV‐SWE relies on two essential conditions: first, capillary action must overcome gravity and successfully initiate siphoning; second, the water driven by capillary action can supply the evaporated water. Figure 2B illustrates the calculated capillary rise of the feed water in the fabric as a function of the water contact angle (*θ*) on the yarn material and the pore side length (*a*). The results indicate that a capillary water column height exceeding 15 cm can be consistently achieved, provided the fiber diameter remains below 0.18 mm and the contact angle is less than 40°. These conditions are satisfied by most cotton fabrics [52]. It is therefore recommended that the highest point of the evaporator, fabricated from cotton fabric‐based material, be positioned less than 15 cm above the water surface. Figure 2C shows the water permeability (*K*) related to the fiber diameter (*d*) and porosity (*ε*). For a typical cotton fabric, the magnitude of the *K* is between 10^−11^ and 10^−7^ m^2^. Based on this, the siphon velocity under different height differences was calculated. As shown in Figure 2D, the velocity shows a positive correlation with both the water permeability (*K*) and the height difference (*h*). Even at very low hydraulic conductivity, a height difference of approximately 1.6 m is sufficient to generate a siphon velocity greater than 0.25 mm/s. Using this minimum velocity as an upper limit, the maximum cooling flux achievable by a 3‐mm‐thick cotton evaporator at siphon velocities below 0.25 mm/s was further evaluated. As shown in Figure 2E, except for extremely low velocity (< 0.15 mm/s) and ultralow porosities (< 0.15), the calculated maximum evaporative cooling flux significantly exceeds the typical heat flux of 1000 W/m^2^ generated by a PV panel under standard illumination. Therefore, these theoretical calculations demonstrate that as long as the highest point of the evaporator does not exceed the maximum capillary height above the water surface, common cotton fabrics in practice can sustain continuous evaporation without drying out after the siphon is initiated.

#### 2.2.2 Steady‐State Characteristics

**FIGURE 3:**
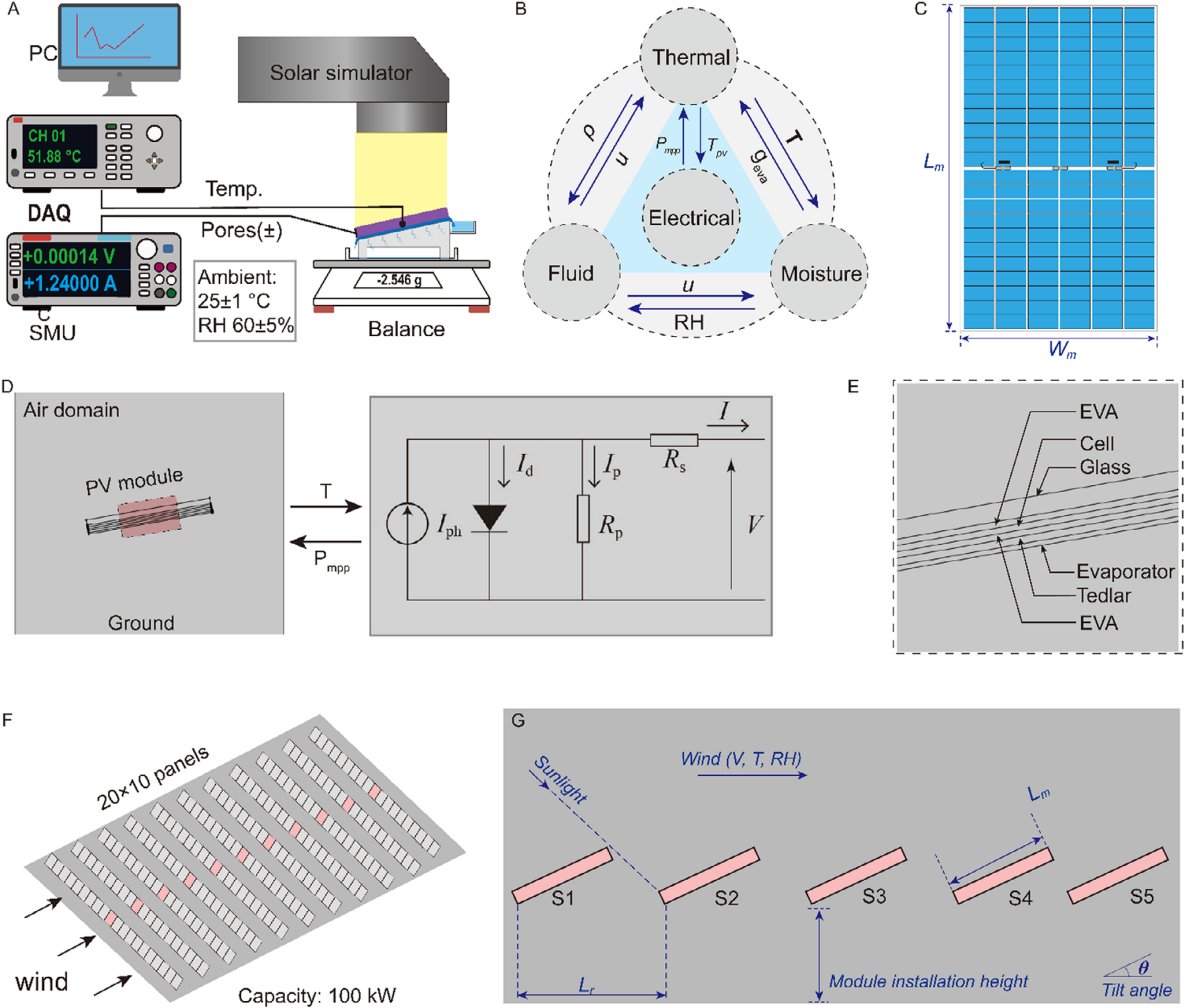
PV‐SWE multiscale methodology. (A) In‐lab experiment setup. (B) Multiphysics coupling strategy. (C) Corresponding schematic diagram of the array layout. (D) 2‐D multiphysics simulation model depicting the array setup. (E) Key parameters for PV module installation. (F) 2‐D multiphysics simulation model depicting the array setup. (G) Key parameters for PV module installation.

Based on the preceding analysis, experiments under wind‐free conditions are performed with a PV‐SWE setup that incorporates a 3‐mm evaporator. Figure 3A shows the schematic of the experiment setup, and its photo is shown in Figure S1. A multiphysics modeling framework to optimize the PV‐SWE performance is developed (detailed in the next section). We use the framework to investigate the temperature and humidity field generated during evaporation. As indicated in Figure 2F, a notable observation is the initial formation of a vapor plume (after 3 min) surrounding the PV‐SWE, which coalesced into a stable structure as evaporation progressed (after 5 min). Concurrently, a thick moisture boundary layer (MBL) developed at the bottom of the evaporator, characterized by a steep humidity gradient from saturation at the interface to the ambient level. A stable temperature field also emerged with increased illuminated duration (Figure 2G). MBL constitutes the crucial zone where water vapor, having absorbed PV waste heat, enters the mainstream flow. Hence, the facilitation of vapor transfer into the mainstream air is paramount to achieving optimal PV‐SWE operation. Subsequent analysis reveals that this MBL is the dominant factor affecting PV‐SWE performance.

### 2.3 PV‐SWE Module Optimization

Based on scaled‐model experimental data, a multiphysics model was constructed for the multi‐scale optimization of commercial‐scale PV‐SWE systems and arrays (Note S1 and Table S1). It allows for a detailed simulation of the PV‐SWE and its local air environment, accounting for conjugate heat transfer, moisture transport, fluid dynamics, and the internal electrical behavior of the PV cell (Figure 3B). As is shown in Figure 3C, the module‐level simulation is modeled using a standard 144‐cell PV panel (*L*_m_: 2234 mm, *W*_m_: 1134 mm). The electrical characteristics are shown in Table S2 and Figure S3. The coupling architecture between the multiphysics and electrical models is presented in Figure 3D, and a detailed view of the PV laminate structure with the evaporator is shown in Figure 3E. For PV‐SWE array, to facilitate the analysis of the electrical and thermal performance distribution across the PV array, several assumptions are made regarding the PV panels. These include: all PV panels being of the identical model, each column of panels operating under uniform illumination, and all panels functioning at the maximum power point tracking (MPPT) state. Furthermore, given that the support structures are generally slender and lightweight, they can be neglected in the modeling process to reduce computational load. Under these reasonable assumptions, a two‐dimensional model consisting of 5 module strings (S1‐S5) of the PV array is developed, as shown in Figure 3F. This model will analyze the impact of both PV array parameters and operational environment conditions. Key array parameters include the tilt angle (*θ*), row spacing (*L*_r_), and ground clearance (*L*_g_).

The accuracy of the model was subsequently validated using a scaled‐down experimental platform. The experimental data included the temperature of the PV panel, the Current‐voltage（*I*–*V* ）curves, measured by a source meter unit after reaching steady‐state temperature, and the water consumption calculated by a mass balance. Good agreements are obtained between the predicted results and experiment (Figure S2). The absolute error in temperature was 1.2 °C, and the *I–V* curves exhibited a correlation coefficient of 0.984. Meanwhile, the predicted weight variation curve closely matched the measurements from the mass balance, with an absolute error of only 119 mL/m^2^ (relative error < 8.4%). These results collectively confirm the validity of the developed model, establishing it as a reliable tool for the design and performance prediction of PV‐SWE systems.

#### 2.3.1 Operating Conditions

**FIGURE 4:**
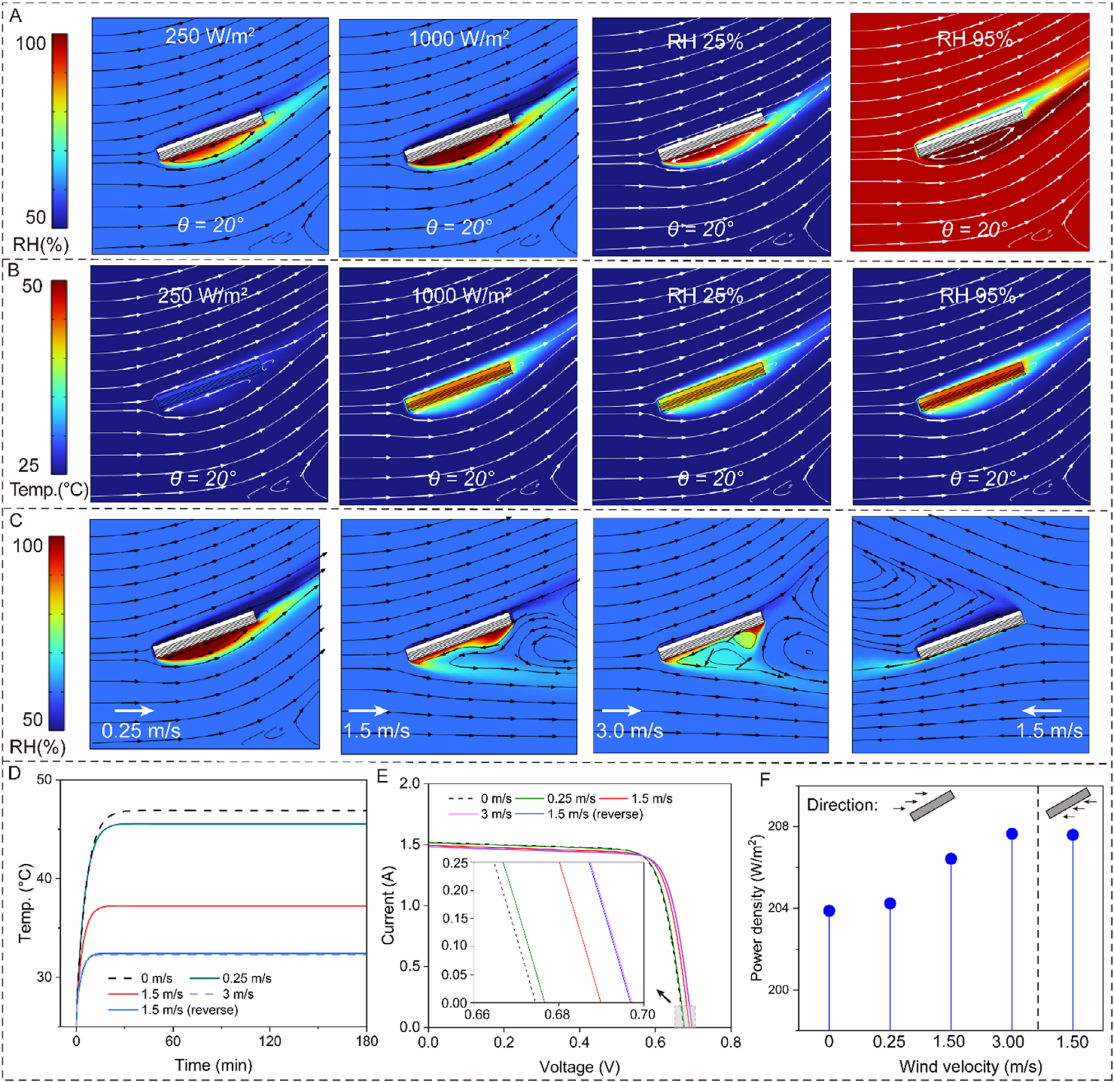
Effect of operating conditions on PV‐SWE performance. (A,B) Temperature field and humidity field under different incident irradiance and RH. (C) Humidity field under different wind velocities. (D) Temperature profiles of the PV‐SWE under different wind velocities. (E) *I–V* curve comparisons under different wind velocities. (F) Power output density under different wind velocities and directions.

As indicated in two panels on the left in Figure 4A,B, the PV operating temperature exhibits a direct correlation with radiation intensity (25 °C, 60% RH, 0.2 m/s breeze). While the distribution patterns are similar across cases, the high‐radiation condition features a markedly thicker saturated region (dark red), indicating a thickened MBL due to intensified evaporation. Comparing results at different relative humidity levels (25 °C, 1000 W/m^2^, 0.2 m/s) shows that higher ambient RH also expands the MBL (two panels on the right in Figure 4A,B). This is attributed to the diminished humidity gradient into the mainstream, which consequently inhibits the evaporation rate.

#### 2.3.2 Effect of Wind Condition

Given the observed strong coupling between near‐evaporator flow and heat transfer, particularly the correlation between MBL growth and PV thermal state under natural convection, we evaluated the impact of forced convection wind fields on the PV‐SWE system. As visible in Figure 4C, airflow effectively disturbs the MBL structure. Figure 4C demonstrates airflow perturbing the MBL on the leeward side of the evaporator, where a higher wind speed thins the layer, resulting in better cooling. Figure 4D reveals that the PV temperature's transient evolution is similar under different wind speeds; however, the final steady‐state value is critically determined by the flow regime. Natural convection (0 and 0.25 m/s) resulted in high operating temperatures of 46.8 °C and 45.5 °C, respectively. In contrast, the establishment of forced convection at wind speeds above 1.5 m/s effectively reduced the temperature below 40 °C. The superior cooling finally boosts the open‐circuit and this enables a peak power output of 208 W/m^2^, as indicated in Figure 4E,F.

#### 2.3.3 Installation Parameters

Next, we examine the effect of the PV tilt angle, a fundamental installation parameter, on the PV‐SWE's electro‐thermal performance (at 1000 W/m^2^, 25 °C, 60% RH, and 2 m/s frontal wind). Figure S4A demonstrates that effective cooling occurs across a range of angles, with the optimum observed at 10° with an operating temperature of 32.5 °C. Consequently, the 10° tilt angle also yielded the highest open‐circuit voltage (Figure S4B), resulting in a power density of 207.8 W/m^2^ (Figure S4C). Figure S4D–F illustrates the mechanism behind the improvement. Superior performance is ascribed to the geometry at this low tilt angle, which allows the oncoming wind to most effectively perturb the MBL structure.

#### 2.3.4 Transient Performance Comparison

Based on the parametric analysis above, we conducted a performance comparison under real dynamic weather conditions, selecting typical summer weather parameters for Hong Kong and fully accounting for the dynamic characteristics of irradiance and the transient effects of wind speed. Figure S5A presents the air temperature and solar irradiance, while Figure S5B shows the relative humidity and wind speed. The meteorological data indicate that although the peak irradiance occurs in the morning, it exhibits unstable and fluctuating characteristics. In contrast, the irradiance in the afternoon is relatively stable with higher wind speeds, while the relative humidity remains high throughout the day. As shown in Figure S5C, after sunrise, the cooling effect of PV‐SWE becomes effective. The variation in operating temperature of the PV module generally follows the trend of solar irradiance, but the cooling effect is influenced by the combined effects of irradiance and wind speed. Overall, under sustained high irradiance, increased wind speed helps maximize the net power output gain of PV‐SWE (Figure S5D). In this case study, the maximum temperature reduction of 22.3 °C occurred at 14:06.

### 2.4 PV‐SWE Array Optimization

Array optimization aims to address scaling challenges for PV‐SWE (Note S2). To balance computational efficiency and accuracy, a mesh‐independent analysis is conducted (Figure S2). The tuned framework first explores how design parameters affect array operation, specifically the cooling performance and power gains from various design patterns, with effects quantified against standard uncooled PV. Then, we also examine electrical string characteristics for potential voltage mismatch due to cooling.

#### 2.4.1 Array Design Parameters

**FIGURE 5:**
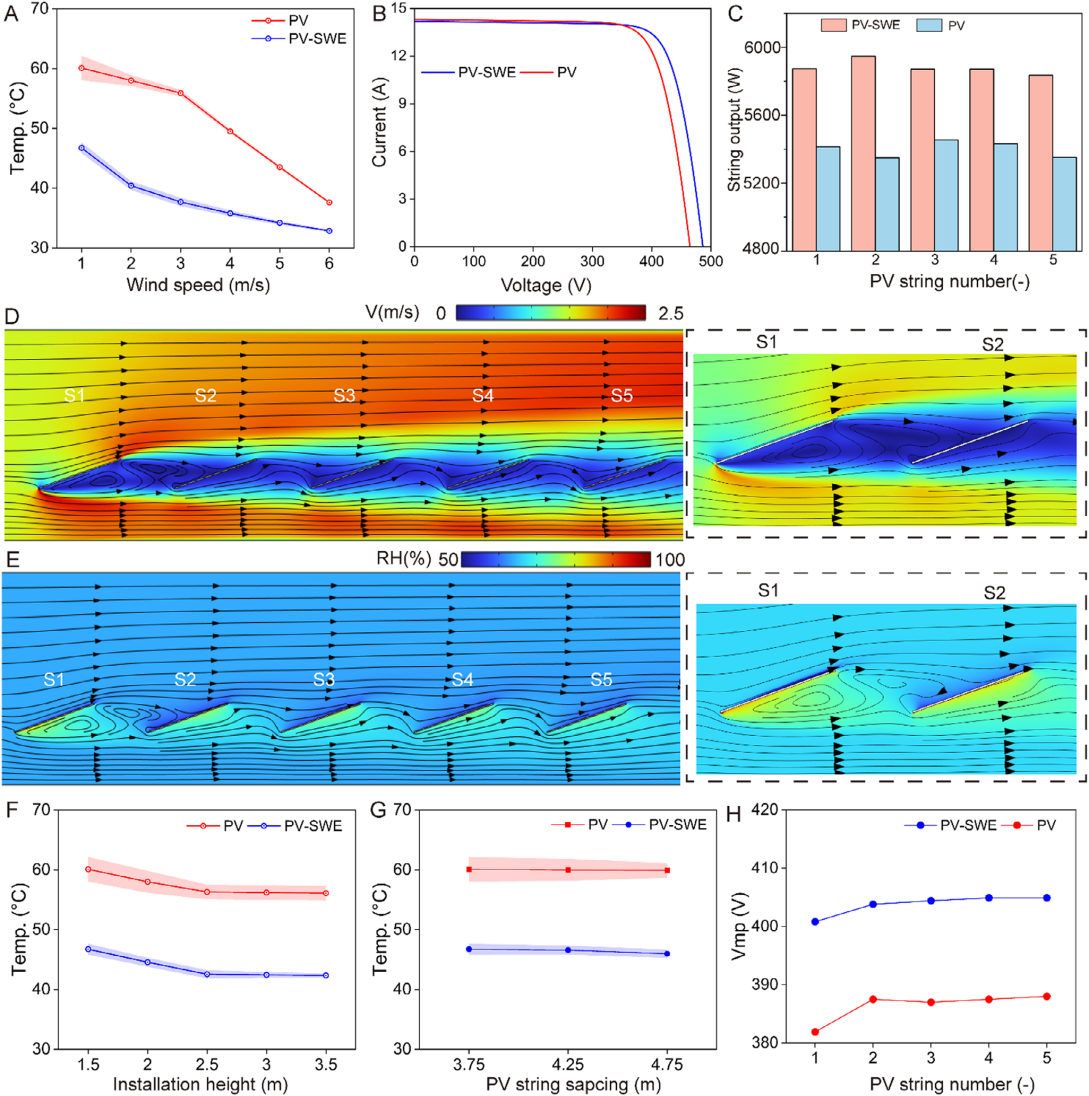
Array‐level optimization of PV‐SWE. (A) The effect of wind speed on the array temperature, with shaded areas representing the standard deviation of temperature. (B) *I–V* curve comparison of normal PV array and PV‐SWE array. (C) Power output comparison. (D) Velocity field of the PV‐SWE array. (E) RH distribution of the PV‐SWE array. (F) Effect of installation height on the array temperature, with shaded areas representing the standard deviation of temperature. (G) Effect of row spacing on the array temperature, with shaded areas representing the standard deviation of temperature. (H) Operating voltage of each module string at MPP state.

The impact of different incoming wind speeds on both conventional PV arrays and PV‐SWE arrays is studied, with the aim of quantifying the cooling effects. The row spacing (*L*_r_) between PV module strings was set at 3.75 m, the minimum distance required to prevent mutual shading between adjacent modules with a tilt angle of 20°. As shown in Figure 5A, wind speed plays a significant role in reducing the operating temperature of module string. Even without evaporative cooling, temperatures can drop significantly below 50 °C when wind speeds exceed 3.4 m/s. When evaporative cooling is applied to the PV array, the operating temperature can be further reduced under the same wind speed conditions. At wind speeds above 3 m/s, the temperature can be lowered to below 40 °C. Generally, PV‐SWE can reduce the PV temperature up to 19 °C. Figure 5B,C shows the electrical performance benefited from cooling, which will be discussed in the next section.

An interesting observation is that the temperature uniformity across the PV strings varies with wind speed. The shaded areas in Figure 5A represent the temperature standard deviation across the entire PV array. At lower wind speeds, the maximum temperature deviation of 4.1 °C is observed. By contrast, the temperature profile becomes more uniform across the array at higher speeds (Figure 5A). This phenomenon can be reasonably explained by examining the velocity and humidity fields. As indicated in Figure 5D,E, the obstruction by the first module string not only significantly impedes the incoming wind, leading to a stagnation zone, but also increases the wind turbulence. Consequently, the downstream PV panels experience a higher proportion of tangential flow despite a lower overall wind speed. This increased tangential flow is more conducive to enhancing the evaporation process. This effect is distinct at low wind speeds but becomes mitigated at high speeds due to the robust airflow, which ensures adequate flow conditions even behind the first row.

The influence of the module string installation height on cooling performance is also investigated. As shown in Figure 5F, it was found that appropriately increasing the height above the ground can effectively enhance evaporative cooling. Compared to the standard height of 1.5 m, raising it to 2.5 m under identical operating conditions (wind speed 1 m/s, 30 °C, and RH50%) resulted in a temperature reduction of 4–5 °C. This improvement is attributed to the more unrestricted airflow from the ground level, which can more readily reach the back of the PV modules, thereby promoting evaporation. However, the benefits exhibit diminishing returns once the height exceeds 2.5 m, as the drag effect from the ground surface on the airflow beneath the panels becomes significantly weaker at greater elevations. Further simulation (wind speed 1 m/s, 30 °C, and 50%) on the row spacing (Figure 5G) between PV strings reveals that although increasing the distance can slightly improve cooling efficiency, the effect is limited. This is primarily because a larger spacing does not effectively enhance the tangential airflow over the evaporative units, which is essential for promoting evaporation. However, this approach comes at the cost of significant land resource consumption. Thus, blind pursuit of greater height should be avoided.

#### 2.4.2 Electrical Characteristics of Array

Cooling finally benefits the electrical performance of the PV array. Figure 5B shows the *I–V* curve comparison of the first module string. As indicated, cooling significantly improves the open‐circuit voltage, with PV string of 463 V while PV‐SWE string reaches 486 V. This improvement significantly improves the PCE of the module string by 8.9% relatively. As a result, the 5 module strings can have an additional 1200 W output. Given that the series connection of PV modules multiplies the voltage, particular attention must be paid to electrical mismatches between strings, which are known to cause a deviation in the maximum power point. As indicated in Figure 5H, although the output of the different module strings is slightly different, with a variance of 50 W, the operating point to obtain the maximum output is nearly the same. The PV‐SWE exhibits a higher Maximum Power Point (MPP) than the standard PV module string; however, the inter‐string voltage difference remains small. This implies that by performing maximum power point tracking (MPPT) on each string, potential mismatch losses can be avoided.

### 2.5 Multi‐Scale Optimization Strategy

Based on the above results, the entire optimization process for the large‐scale application of PV‐SWE can be summarized as follows. First, ensure that the distance between the top of the evaporator and the water surface is less than the maximum capillary water column height that the material can provide, thereby guaranteeing the smooth initiation of the siphon process and enabling passive water transport. On this basis, the installation process should ensure adequate ventilation at the back of the evaporator, thereby promoting the disruption of the boundary layer by the incoming wind. For utility‐scale applications, there is no need to increase the spacing between module strings. Instead, an installation height of 2.5 m is sufficient to reduce the drag effect of the ground on the wind and achieve effective cooling.

## 3 Conclusion

This study proposes an efficient PV cooling technology based on interfacial evaporative cooling. It enables passive operation via a siphon‐driven water supply and effectively dissipates waste heat through evaporation. Using a multiscale methodology of numerical simulation and experiment, we evaluate the application potential of this technology. A prototype is developed based on theoretical calculations. A modeling framework elucidating the internal heat and moisture transfer mechanism is developed and demonstrates high accuracy, with a temperature prediction error below 0.7 °C, an *I–V* curve correlation coefficient of 0.984, and a weight change error of 119 mL/m^2^. Passive siphon requires the evaporator top to be within the material's capillary height (below 14 cm for cotton fabric). A 3‑mm cotton‑based evaporator with a siphon speed of 0.25 mm/s provides a cooling flux exceeding 2000 W/m^2^, far surpassing PV heat loads. By optimizing the installation angle to exploit wind disruption of the moisture boundary layer, a temperature reduction of about 22.3 °C can be achieved, which significantly outperforms existing PV cooling technologies. For utility‑scale implementation, existing PV plant layouts can be retained without enlarging row spacing. Raising installation height improves cooling, but gains diminish beyond 2.5 m. The system exhibits excellent cooling uniformity (maximum temperature difference ≤ 4 °C) and boosts PV efficiency by 8.9% without electrical compatibility issues. This work clarifies key mechanisms and operational factors, offering practical guidance for real‑world PV cooling applications.

## 4 Materials and Methods

### 4.1 Experimental Section

#### 4.1.1 Materials

PV glass was supplied by Suzhou Mechatronics. Solar cells, EVA, and Tedlar were supplied by Misuole New Energy. The 3 M 467MP thermally conductive double‐sided adhesive tape was bought from the 3J as our TIM. Cotton fabric was purchased from Kee.

#### 4.1.2 Module Fabrication

A monocrystalline silicon solar cell (SunPower C60) is wired and fabricated into a small PV module for measurement. The fabrication follows the lamination process: low‐Fe glass, 2 EVA layers, wired solar cell, and Tedlar sheet are stacked and laminated at 120–140 °C under a vacuum of −1 MPa for 15–20 min.

#### 4.1.3 PV‐SWE Cooling Experiment

A SciSun‐300 Solar Simulator (Class AAA, AM 1.5 filter) with a calibrated reference cell was used to provide simulated sunlight. An electronic balance (ME503T, Mettler Toledo) was used to measure the evaporation rate. A Keithley‐2460 source meter was used to measure the *I–V* and *P–V* curves of the solar cell. The Keysight DAQ970A data acquisition system was used to record the temperature. Both the room temperature and RH were controlled by a heating, ventilation, and air‐conditioning (HVAC) system. The room temperature was maintained at 25 °C, and the RH was maintained at 60%.

#### 4.1.4 Characterization

The morphology of the wicking evaporator was characterized by a scanning electron microscope (EVO MA10, Zesis). The contact angles were measured by an optical contact angle meter (Theta Flex, Biolin) at an ambient temperature of 25 °C using a 5 mL water droplet as the indicator.

### 4.2 Theoretical Calculation

#### 4.2.1 The Initiation of the Siphon Flow

The highest point (*h*) of the evaporator must be adjusted based on the attainable capillary height (*h_c_*), as this is fundamental to the entire system. The height of the capillary water column *h_c_* is determined by the structural properties of the evaporator material and the contact angle between the evaporator material and water. For hydrophilic fabrics like cotton, the fiber diameter and pore size can be used to describe their structure. These parameters allow for the calculation of the capillary height *h_c_* in such materials [53].

(1) $$h_{c}=\frac{\left(2+\sqrt{\pi }\right)\sigma \cos\theta }{\rho ga}$$

where *h_c_* is water column height, m; σ is the surface tension of the liquid, N/m; θ is the contact angle of the liquid on the evaporator material, deg; ρ is the water density, kg/m^3^; *g* is the gravitational acceleration speed, m/s^2^; *a* is the side length of the pore, mm.

#### 4.2.2 Performance Limit of SWE

Following the start of siphon flow, the second condition is that the evaporative heat flux (*Q*_eva_) should be greater than the PV heat load (*Q*_PV_). In the case of a porous thin‐film evaporator, the internal flow process after siphon formation can be modeled via Darcy's law.

(2) $$v=\sqrt{\left(2\left(gh\cos\theta -\frac{\Delta P}{\rho }\right)\right)}$$

where *v* is the water velocity, m/s; *L* is the flow distance, which is the module length here, m; Δ*P* is the pressure drop caused by flow resistance, Pa; ρ is the water density, kg/m^3^; *g* is the gravitational acceleration speed, m/s^2^.

The pressure drop can calculate using the following equation:

(3) $$\Delta P=\frac{v\mu L}{K}$$

where *K* is the permeability of the porous medium, m^2^; μ is the dynamic viscosity of the fluid, Pa·s.

*K* can be estimated using the Kozeny–Carman equation [54]:

(4) $$K=\frac{\varepsilon^{3}d^{2}}{180{\left(1-\varepsilon \right)}^{2}}$$

where ε is the porosity of the material; *d* is the effective particle size (taken as the fiber diameter for a fabric), mm.

By solving the above equations, the flow velocity *u* can be calculated and which enables an approximation of the mass flow rate achievable, thereby determining the maximum cooling capacity *Q_eva_*:

(5) $$Q_{eva}=\rho uAH_{eva}$$

where *A* is the cross‐sectional area, m^2^; *H_eva_* is the evaporation enthalpy of the water, kJ/kg.

#### 4.2.3 PV‐SWE Modeling Framework

The modeling framework is elaborated in Note S1.

## Funding

National Natural Science Foundation of China (Nos. 52322812, and 52476019), the Research Grants Council of Hong Kong (No. CityU 11218922), and the Conservation Fund of Hong Kong (No. 76/2022), and City University of Hong Kong (No. 9667263).

## Conflicts of Interest

The authors declare no conflicts of interest.

## Supporting information




**Supporting File**: advs75120‐sup‐0001‐SuppMat.docx.

## Data Availability

The data that support the findings of this study are available from the corresponding author upon reasonable request.
