# Supplementary material for: Multi‐scale Optimization on Interfacial Evaporative Cooling for Photovoltaic Performance Enhancement
Source: Adv Sci (Weinh). 2026 Apr 7;13(34):e75120. doi: 10.1002/advs.75120 (PMC13285113; doi:10.1002/advs.75120)
Supplement: Supplementary file 1 — Supporting File: advs75120‐sup‐0001‐SuppMat.docx. [file ADVS-13-e75120-s001.docx]

Supporting Information

Multi-scale Optimization on Interfacial Evaporative Cooling for Photovoltaic Performance Enhancement

Fuxiang Li^1^**,**^2^, Haosheng Lin^1^**,**^2^, Zengguang Sui^1^**,**^2^, Yunren Sui^1^**,**^2^, Zhixiong Ding^1^**,**^2^, Wei Wu^* 1^**,**^2^

1 School of Energy and Environment, City University of Hong Kong, Hong Kong, China

2 Shenzhen Research Institute, City University of Hong Kong, Shenzhen, China

*Corresponding author: Wei Wu

Email: [weiwu53@cityu.edu.hk](mailto:weiwu53@cityu.edu.hk)

Supplemental Figures


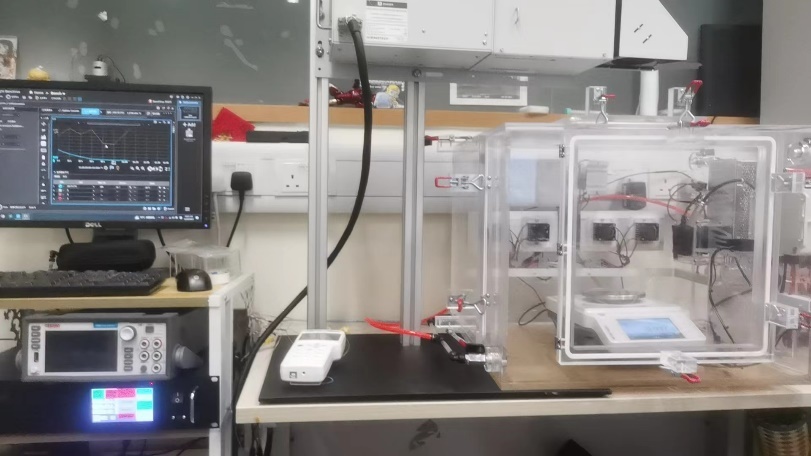


**Figure S1**. Photo of the experiment setup.


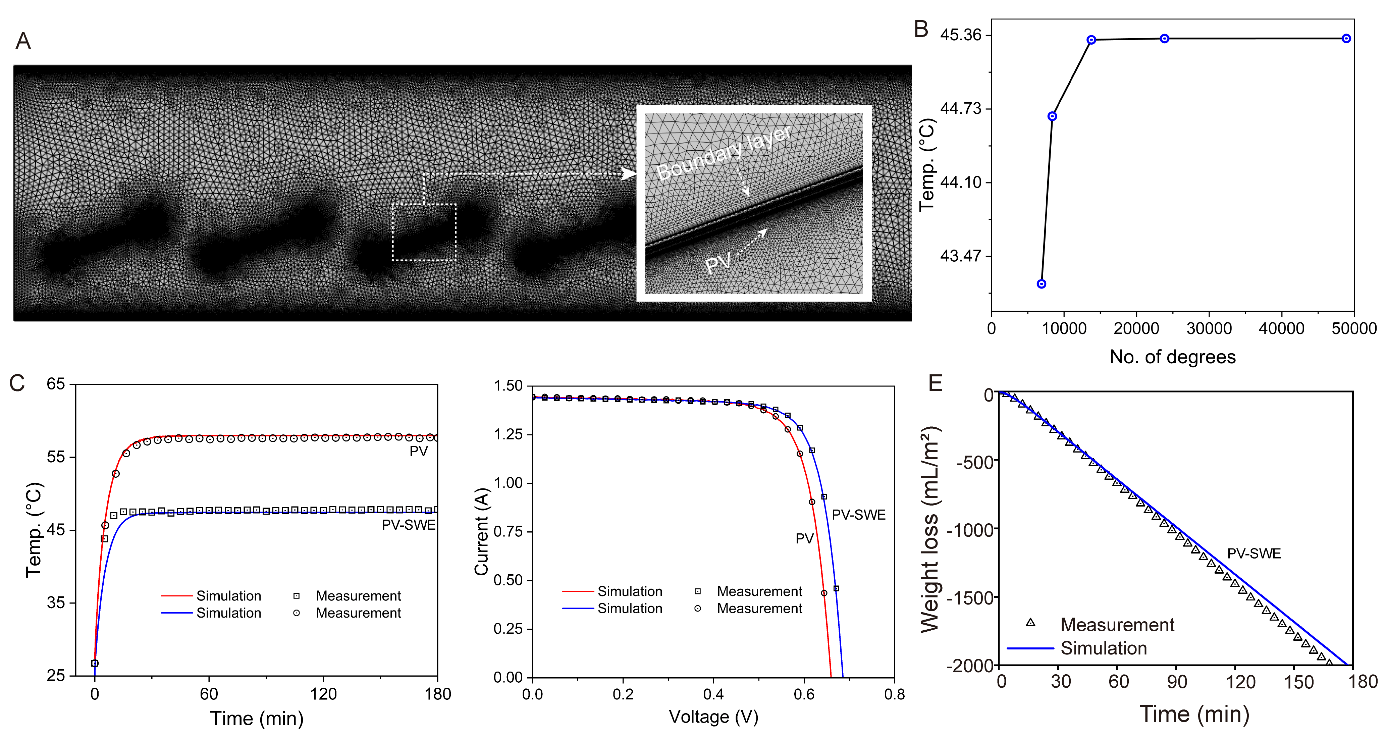


**Figure S2.** Mesh independence analysis and model validation demonstrate that the developed framework can accurately predict both the electrical and thermal performance of the PV-SWE, as well as that of the PV without cooling. (A) Mesh configuration. (B) Mesh independence study. (C) Comparison of predicted and measured temperature. (D) Predicted I-V curve vs. measured I-V curve. (E) Predicted weight changes vs. measured weight changes.


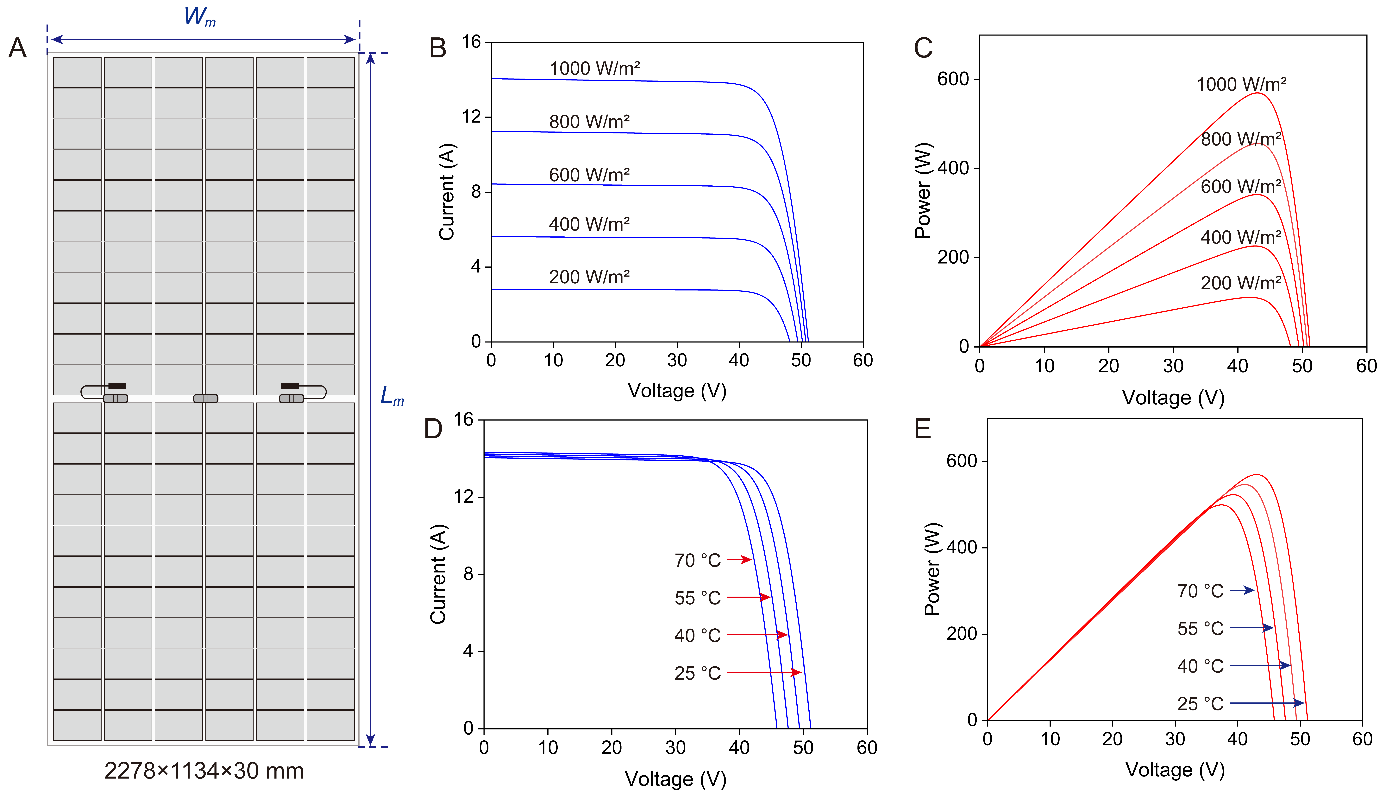


**Figure S3.** PV-Module geometry parameter and electrical characteristics. (A) Module structure diagram. (B-C) I-V and P-V curves under different irradiance with the same temperature of 25 °C. (D-E) I-V and P-V curves with various temperatures under the same irradiance of 1000 W/m^2^.


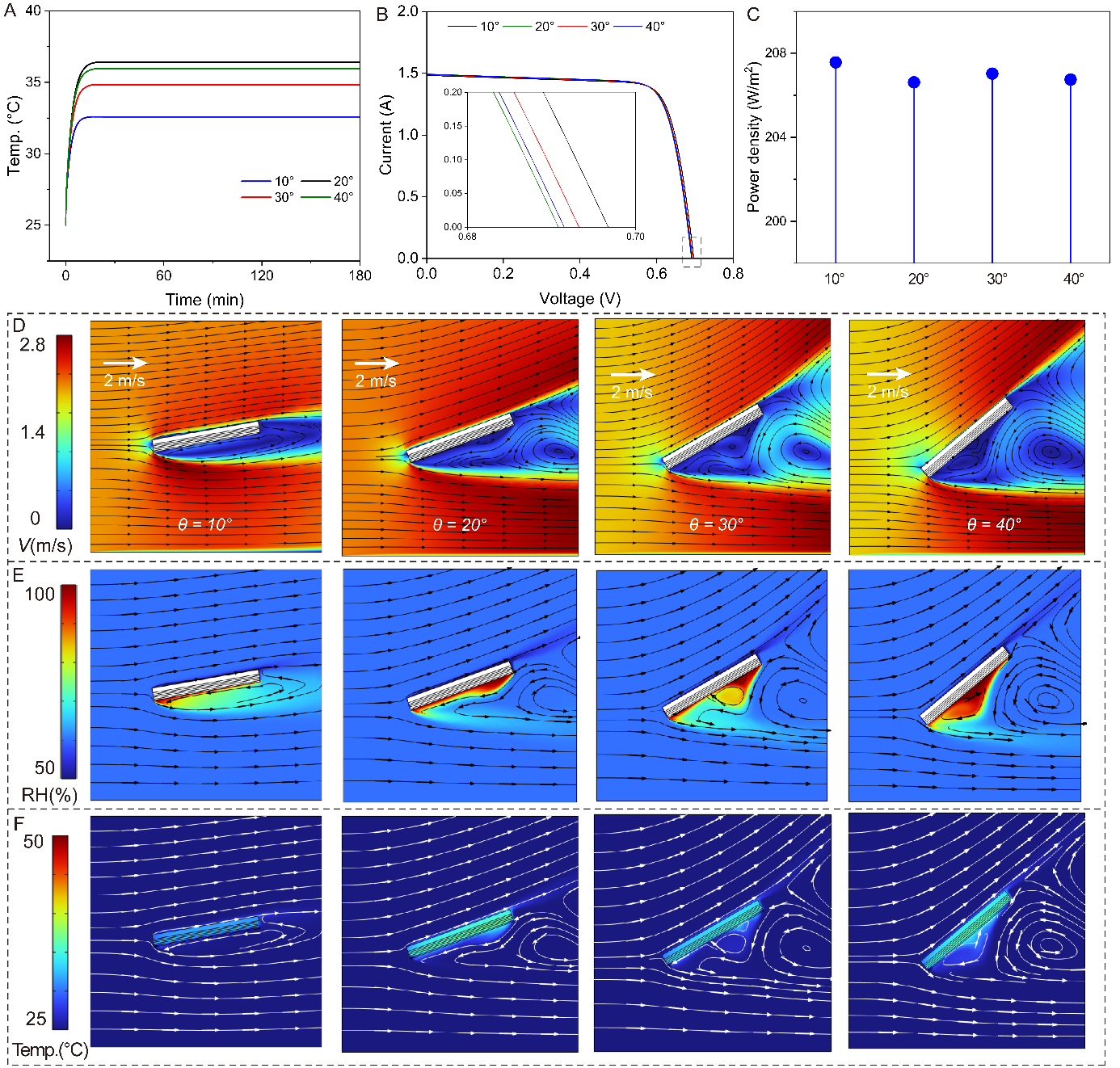


**Figure S4.** Effect of incident tilt angles on PV-SWE performance. (A) Temperature profiles of the PV-SWE. (B) I-V curve comparisons. (C) Power output density of PV-SWE. (D) Velocity field. (E) Relative humidity field. (F) Temperature field.
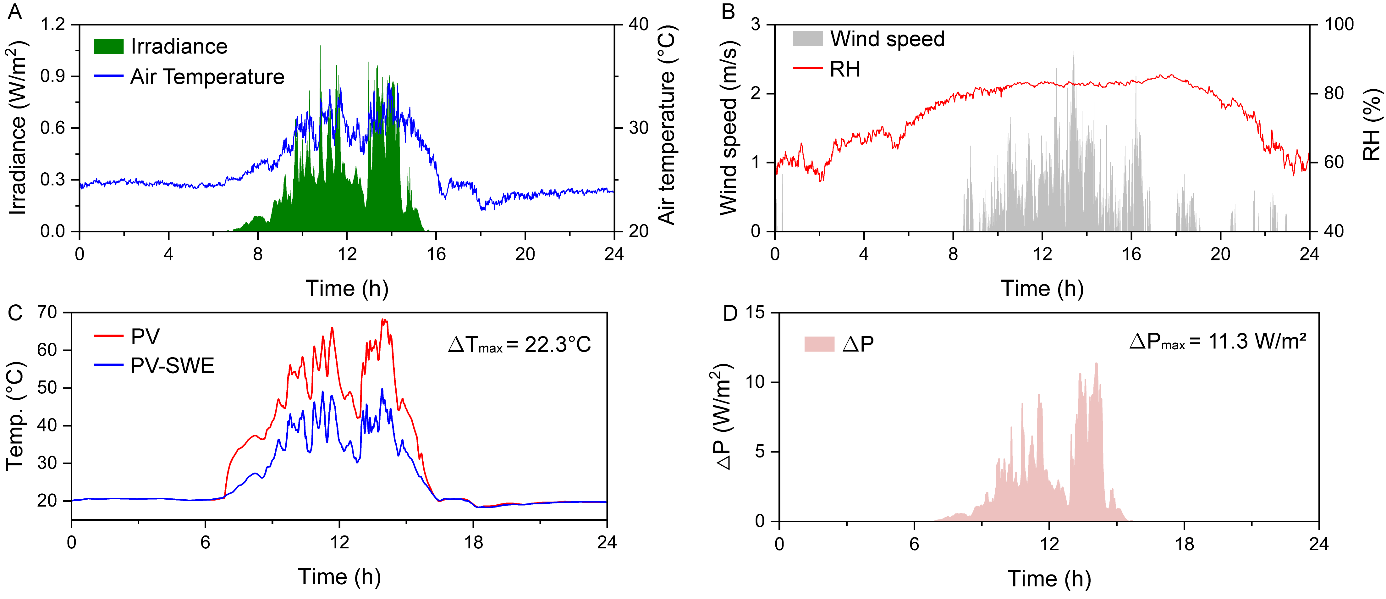


**Figure S5.** Transient performance comparison of PV and PV-SWE under typical summer weather conditions. (A) Irradiance and air temperature profiles. (B) Wind speed and humidity profiles. (C) Operating temperature comparison between PV and PV-SWE. (D) Net power output gain due to cooling.


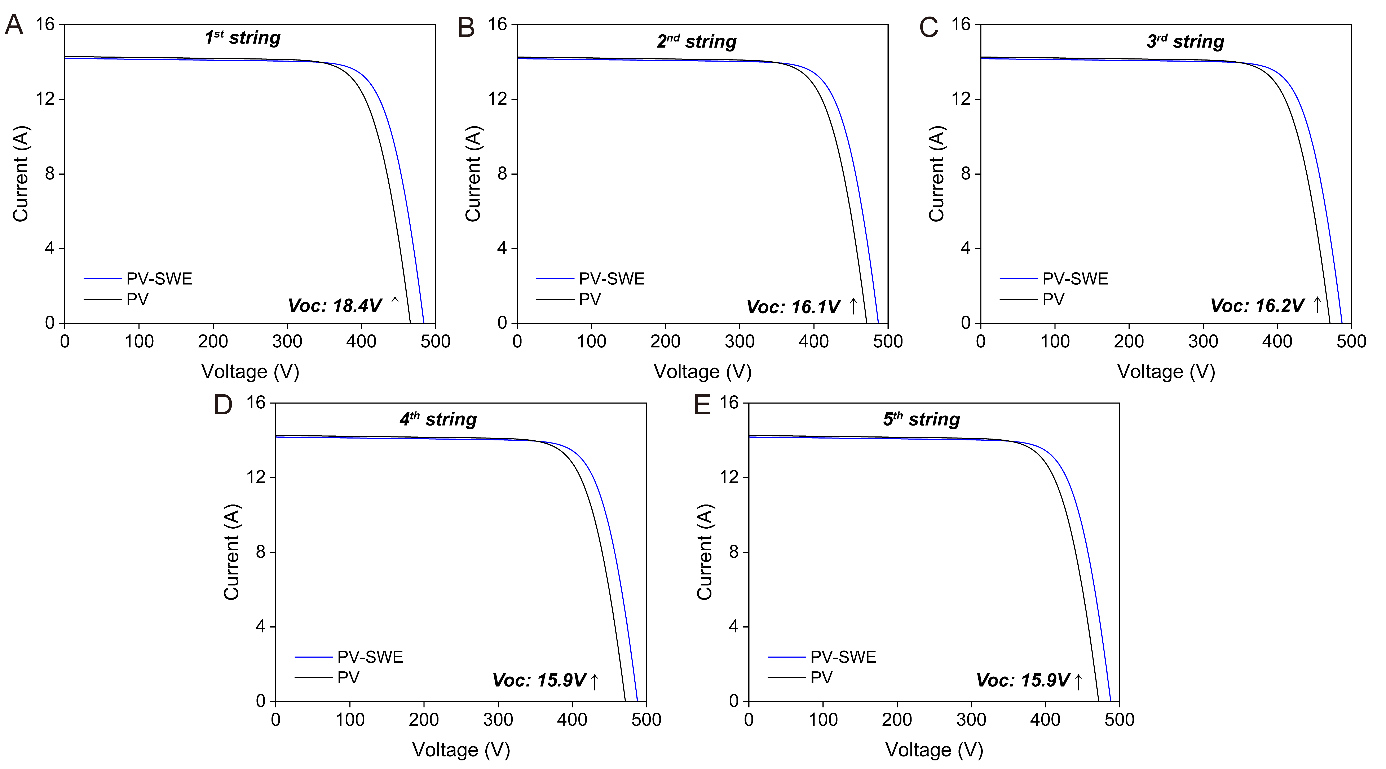


**Figure S6.** Array-level electrical performance comparison of PV and PV-SWE. (A) 1^st^ module string. (B) 2^nd^ module string. (C) 3^rd^ module string. (D) 4^th^ module string. (E) 5^th^ module string.

Supplemental Notes

Note S1. PV-SWE modeling framework

A central feature of module-level modeling framework is the representation of key couplings: electricity generation within the PV cell; the interplay of heat transfer, capillary flow and evaporation in the evaporator; and the conjugate heat and mass transfer with the ambient moist air.

1. **Energy conservation**

The energy conservation in the PV-SWE mainly encompasses three domains: the PV module, the evaporator and the surrounding air. For the PV module domain, the energy conservation process can be regarded as a heat transfer problem in pure solid phase. The PV cell layer acts as a solar absorbing layer in which the incident sunlight is partially converted into electricity by PV cell and the rest are converted into heat. This photovoltaic process will be two-way coupled with an electrical model detailed in the following section. Thus, the governing energy conservation equation is expressed as:

$\rho c\frac{\partial T}{\partial t}-\nabla\cdot(\lambda\nabla T)=0$ (Equation S1)

where $\rho$ is the density, kg/m^3^; $c$ is the specific heat capacity, J/(kg∙K); $T$ is the temperature, K; $\lambda$ is the thermal conductivity, W/(m∙K).

For the SWE domain and surrounding air, the influence of flow processes on heat transfer must be accounted for. The former involves the flow of moisture within the evaporator, while the latter pertains to the flow within moist air. This requires different equations to calculate the velocity field for the two phases, respectively. However, the governing energy conservation equations are the same:

$\rho c_{p}\frac{\partial T}{\partial t}+\rho c_{p}\text{u}\cdot\nabla T-\nabla\cdot\left(k\nabla T\right)=0$ (Equation S2)

where $c_{p}$ is the specific heat capacity, kJ/kg; $\text{u}$ is the velocity vector, m/s; $k$ is the thermal conductivity, W/(m·K).

1. **Fluid dynamic model**

The moisture flow within the SWE under gravity and the disturbance from the surrounding air impact the overall performance of the PV-SWE. The former can be described using Darcy's law:

$\frac{\partial}{\partial t}\left(\varepsilon\rho\right)+\nabla\cdot\left(\rho\text{u}\right)=0$ (Equation S3)

$\text{u}=-\frac{\kappa}{\mu}\left(\nabla p-\rho g\right)$ (Equation S4)

where $\varepsilon$ is the porosity of the evaporator; $\rho$ is the density of the water, kg/m^3^; $\text{u}$ is the velocity field of the water inside the evaporator, mm/s.

The air domain is described by the continuity equation and time-dependent compressible Navier-Stokes equations:

$\rho\frac{\partial\text{u}}{\partial t}+\nabla\cdot\left(\rho\text{u}\right)=0$ (Equation S5)

$\rho\frac{\partial\text{u}}{\partial t}+\rho\left(\text{u}\cdot\nabla\right)\text{u}=-\nabla p+\nabla\cdot\mu\left(\nabla\text{u}+\left(\nabla\text{u}\right)^{T}\right)+\rho\text{g}$ (Equation S6)

where $\rho$ is the density of the moist air, kg/m^3^; $\text{u}$ is the velocity vector, m/s; $p$ is the pressure, Pa; $\mu$ is the dynamic viscosity, Pa∙s; $g$ is the gravity, 9.8 kg∙m/s^2^.

For high wind speed cases and potentially turbulent conditions in array-level simulations, we neglect the compressibility and gravitational effects of the fluid in time-dependent compressible Navier-Stokes equations.

$\rho\nabla\cdot u=0$ (Equation S7)

$\rho\frac{\partial\text{u}}{\partial t}+\rho\left(\text{u}\cdot\nabla\right)\text{u}=-\nabla p+\nabla\cdot\mu\left(\nabla\text{u}+\left(\nabla\text{u}\right)^{T}\right)$ (Equation S8)

The Low Reynolds Number k-ε turbulence model is used to simulate turbulence. The governing equations for turbulent kinetic energy $k$ and turbulent dissipation rate $\varepsilon$ in the turbulence model are as follows:

(Equation S9)

(Equation S10)

The production term $P_{k}$ and turbulence viscosity $\mu_{T}$ is described as:

$P_{k}=\mu_{T}\left(\nabla u:\left(\nabla u+\left(\nabla u\right)^{T}\right)-\frac{2}{3}\left(\nabla\cdot u\right)^{2}\right)-\frac{2}{3}\rho k\nabla\cdot u$ (Equation S11)

$\mu_{T}=\rho f_{\mu}C_{\mu}\frac{k^{2}}{\varepsilon}$ (Equation S12)

The model contains four constants: $C_{\varepsilon1}=1.5$, $C_{\varepsilon2}=1.9$,$C_{\mu}=0.09$,$\sigma_{k}=1.4$ and $\sigma_{\varepsilon}=1.4$. Additionally, $f_{\mu}$ and $f_{\varepsilon}$ are two model functions and they are expressed as:

$f_{\mu}=\left(1-e^{-l^{*}/14}\right)^{2}\cdot\left(1+\frac{5}{R_{t}^{3/4}}e^{-(R_{t}/200)^{2}}\right)$ (Equation S13)

$f_{\varepsilon}=\left(1-e^{-l^{*}/3.1}\right)^{2}\cdot\left(1-0.3e^{-(R_{t}/6.5)^{2}}\right)$ (Equation S14)

The two model functions are primarily determined by the wall distance $l^{*}$, the turbulent Reynolds number $R_{t}$, and the turbulent dissipation velocity $u_{\varepsilon}$. Their calculation formulas are as follows:

$l^{*}=\left(\rho u_{\varepsilon}l_{w}\right)/\mu$ (Equation S15)

$R_{t}=\rho k^{2}/\left(\mu\varepsilon\right)$ (Equation S16)

(Equation S17)

1. **Moisture transport model**

After moisture evaporates from the evaporator, its position changes due to its own concentration gradient and the outdoor airflow. This moisture transport process can be described using the advection-diffusion equation:

$\frac{\partial c}{\partial t}+\text{u}\cdot\nabla c-D\cdot\nabla c=0$ (Equation S18)

where $c_{p}$ is the specific heat capacity, kJ/kg; $k$ is the thermal conductivity, W/(m·K); $c$ is the mole concentration of the moisture, mol/m^3^; $D$ is the diffusivity of the moisture, m/s^2^.

1. **Electrical model**

To quantify the cooling effects on the PV performance, we use a single-diode model (SDM) for photovoltaic process modeling. By solving the SDM, we can accurately predict I-V characteristics of the PV under specific working conditions. It is based on Kirchhoff's law and quantitatively describes the current and voltage relation inside PV modules:

$I=I_{ph}-I_{0}\left[\exp(\frac{V+IR_{s}}{V_{t}})-1\right]-\frac{V+IR_{s}}{R_{p}}$ (Equation S19)

where $I$ is the current in the circuit, A; $I_{ph}$ is the photocurrent generated by the solar cell, A;$I_{0}$ is the saturation current of the diode, A; $V$ is the voltage, V; $R_{s}$ is the series resistance, $\Omega$; $R_{p}$ is the shunt resistance, $\Omega$.

There are five unknown parameters in SDM, which can be determined from the parameters under standard testing (STC) conditions, which can be found in the PV datasheet. These parameters include the short-circuit current ($I_{sc}$), open-circuit voltage ($V_{oc}$), maximum power point current and voltage ($I_{mp}$, $V_{mp}$), as well as the temperature coefficients for voltage ($K_{v}$) and current ($K_{i}$). We use the same parameter extraction method based on our previous work ^[19]^. The datasheet values and extracted parameters are listed in Table S1.

1. **Boundary conditions**

Convective boundary conditions are applied on the sides of the PV module:

(Equation S20)

where *h* is the convective heat transfer coefficient, W/(m^2^·K); *T*_0_ is the ambient temperature, °C.

For the PV cell layer, the boundary heat source is applied to simulate the solar heating effects:

(Equation S21)

where $\alpha$ is the solar irradiance absorption coefficient; $q_{sol}$is the incident sunlight, W/m^2^; $P_{mpp}$ is the maximum power output of the unit PV panel, W/m^2^.

For the front surface, the radiative cooling effect is simulated by using the following equation:

(Equation S22)

where $\sigma$ is the Stefan-Boltzmann constant; $\varepsilon$ is the emissivity of the top surface; $F_{sky}$ is the view factor between the front surface and sky; $T_{sky}$ is the sky temperature.

$T_{sky}=0.0552T_{0}^{1.5}$ (Equation S23)

$F_{sky}=\frac{1+\cos(\beta)}{2}$ (Equation S24)

where $\beta$ is the PV tilt angle.

Pressure boundary conditions are applied at both the top and bottom ends of the evaporator：

$p=\rho gh$ (Equation S25)

where $p$ is the gauge pressure, Pa; $g$ is the gravity, 9.8 kg∙m/s^2^; h is the height relative the ground, m.

The evaporative cooling effect on the membrane surface is:

(Equation S26)

where $h_{l}$ is the latent heat of the water evaporation, J/kg; $J_{eva}$ is the vapor flux between the evaporator and the ambient, kg/(m^2^·s).

1. **Multiphysics coupling strategy**

The fluid-thermal coupling in the moist air domain is primarily manifested as the effect of temperature on air density ($\rho$) and the influence of the velocity field ($u$) on the temperature field during fluid heat transfer. Simultaneously, the latent heat carried away by evaporation ($g_{eva}$) is also simulated through boundary conditions. The water vapor concentration field is likewise coupled with and influenced by the velocity field while the moist air density is also affected by the relative humidity (RH). These couplings are all implemented in COMSOL. Notably, the electrical model is performed using functions in MATLAB and subsequently called within COMSOL with the maximum power output and operating temperature as the coupling variables.

**Supplemental Tables:**

**Table S1** Datasheet values and extracted parameters of scaled down PV-SWE.

| **Datasheet values** | | **Extracted STC parameters** | |
| --- | --- | --- | --- |
| Parameter | Value | Parameter | Value |
| $I_{sc}\left(A\right)$ | 1.46 | $I_{ph}\left(A\right)$ | 1.4671 |
| $V_{oc}\left(V\right)$ | 0.71 | $I_{0}\left(A\right)$ | 7.069×10^-12^ |
| $I_{mp}\left(A\right)$ | 1.34 | $V_{t}\left(V\right)$ | 0.0272 |
| $V_{mp}\left(V\right)$ | 0.60 | $R_{s}\left(\Omega\right)$ | 0.0210 |
| $K_{v}\left(mV/K\right)$ | -1.84 | $R_{p}\left(\Omega\right)$ | 9.5920 |
| $K_{i}\left(mA/K\right)$ | 2.60 |  |  |

**Table S2** Datasheet value of the PV module and extracted parameters for SDM models.

| **STC Parameter** | | **Extracted Parameters** | |
| --- | --- | --- | --- |
| Parameter | Value | Parameter | Value |
| $I_{sc}\left(A\right)$ | 14.05 | $I_{ph}\left(A\right)$ | 14.0619 |
| $V_{oc}\left(V\right)$ | 51.19 | $I_{0}\left(A\right)$ | 1.393×10^-11^ |
| $I_{mp}\left(A\right)$ | 13.26 | $V_{t}\left(V\right)$ | 0.0129 |
| $V_{mp}\left(V\right)$ | 43 | $R_{p}\left(\Omega\right)$ | 205.6382 |
| $K_{v}\left(mV/K\right)$ | -118 | $R_{s}\left(\Omega\right)$ | 0.1739 |
| $K_{i}\left(mA/K\right)$ | 6.3 |  |  |
